# Iron-sulfur cluster-dependent enzymes and molybdenum-dependent reductases in the anaerobic metabolism of human gut microbes

**DOI:** 10.1093/mtomcs/mfae049

**Published:** 2024-11-05

**Authors:** Leah E Zahn, Paige M Gannon, Lauren J Rajakovich

**Affiliations:** De partment of Chemistry, University of Washington, Seattle, United States; De partment of Chemistry, University of Washington, Seattle, United States; De partment of Chemistry, University of Washington, Seattle, United States

**Keywords:** metalloenzyme, iron-sulfur cluster, molybdenum cofactor, human gut microbiota, anaerobic metabolism, redox chemistry

## Abstract

Metalloenzymes play central roles in the anaerobic metabolism of human gut microbes. They facilitate redox and radical-based chemistry that enables microbial degradation and modification of various endogenous, dietary, and xenobiotic nutrients in the anoxic gut environment. In this review, we highlight major families of iron-sulfur (Fe–S) cluster-dependent enzymes and molybdenum cofactor-containing enzymes used by human gut microbes. We describe the metabolic functions of 2-hydroxyacyl-CoA dehydratases, glycyl radical enzyme activating enzymes, Fe–S cluster-dependent flavoenzymes, U32 oxidases, and molybdenum-dependent reductases and catechol dehydroxylases in the human gut microbiota. We demonstrate the widespread distribution and prevalence of these metalloenzyme families across 5000 human gut microbial genomes. Lastly, we discuss opportunities for metalloenzyme discovery in the human gut microbiota to reveal new chemistry and biology in this important community.

## Graphical Abstract

**Graphical Abstract:**
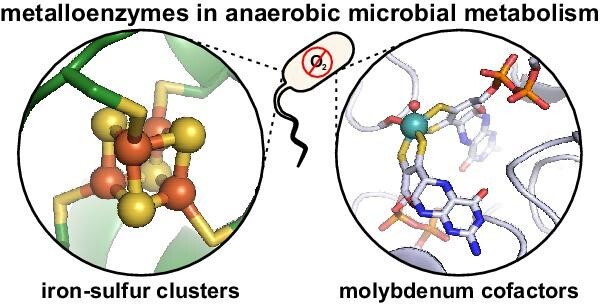

## Introduction

The gut microbiota carries out a wide range of chemical transformations in the human body. This complex microbial community has a vast amount of genetic diversity, collectively encoding for approximately 150-fold more genes than the human genome [1]. Consequently, the microbiota has broad metabolic capabilities to utilize a variety of nutrients from the diet, host, and xenobiotic sources [2–6]. The ability to degrade and modify these molecules provides niche advantages for commensal and pathogenic organisms in this competitive ecosystem.

Gut microbial metabolism contributes substantially to the human metabolome and has important consequences for host health. A recent study estimated that >50% of plasma metabolites are associated with the human gut microbiota [7]. However, the metabolic output of the microbiota is highly variable, depending on an individual's unique microbial composition, as well as host, dietary, and environmental factors [3, 8, 9]. Because microbial metabolites are absorbed and circulated throughout the body [7], they can exert effects on host physiology outside of the gastrointestinal tract. Indeed, numerous intestinal and systemic diseases are correlated with metabolites produced exclusively by the gut microbiota [2, 10–12]. Despite its importance for human health, our understanding of gut microbial metabolism is still limited. Many microbial metabolites have unknown origins, and many microbial proteins have unknown functions [13]. Thus, there is a critical need for biochemical elucidation of metabolic pathways and microbial enzymes to address the impact of the microbiota on host health.

Metalloenzymes play key roles in the anaerobic metabolism of human gut microbes [14]. The architecture of the colon is associated with a radial oxygen gradient, with microoxic conditions near the epithelium and anoxic conditions in the lumen [15]. These oxygen-depleted conditions largely favor anaerobic metabolism, which is often mediated by metalloenzymes. These enzymes catalyze challenging redox and radical-based chemical transformations, essential for degradation and modification of diverse substrates and energy production [14]. In this review, we describe prominent families of metalloenzymes found in human gut microbes. We focus on enzymes that use two major types of redox-active metallocofactors: iron-sulfur clusters and molybdenum cofactors. We describe characterized members of each family that are known to be involved in the unique metabolism of the gut microbiota. Lastly, we profile the distribution of these metallo-enzyme families in ∼5000 human gut microbial genomes and discuss opportunities for discovery of novel metalloenzyme functions in this important microbial community.

## Iron-sulfur cluster-dependent enzymes

Iron-sulfur (Fe–S) clusters are ancient inorganic protein cofactors that are ubiquitous throughout the bacterial kingdom [16]. The two major structural forms are [Fe_2_S_2_] and [Fe_4_S_4_] clusters most commonly ligated by proteinaceous cysteine residues; however, there are rarer instances of other residues (e.g. histidine, serine, aspartate, glutamate) and backbone amides acting as cluster ligands [16–19]. These cofactors are well known for their critical roles in intra- and inter-protein electron transfer [16–20]. In addition, Fe–S clusters serve key catalytic functions in many important enzyme families, particularly in anaerobic bacteria [16, 17]. In this section, we highlight four prevalent families of Fe–S cluster-dependent enzymes that enable many unique metabolic capabilities of the human gut microbiota.

### 2-Hydroxyacyl-CoA dehydratases in amino acid metabolism

**Figure 1.**
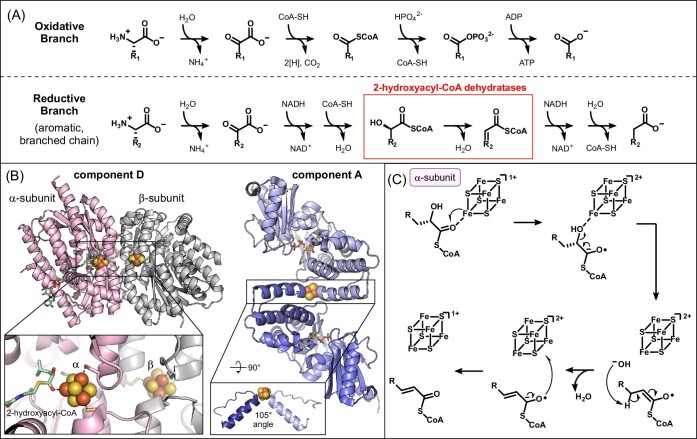
Fe–S cluster-dependent 2-hydroxyacyl-CoA dehydratases in reductive branch of amino acid Stickland fermentation. (A) Oxidative and reductive branches of Stickland fermentation of branched chain and aromatic amino acids (B) X-ray crystal structures of the (*R*)-2-hydroxyisocaproyl-CoA dehydratase component D (PDB 3O3N) and activase component A (PDB 4EHT). Fe–S clusters are shown as spheres, the 2-hydroxyisocaproyl-CoA substrate is shown as green sticks, and two ADP molecules are shown as yellow sticks. (C) Proposed mechanism for Fe–S cluster-mediated dehydration of 2-hydroxyacyl-CoA substrates, occurring in the active site of the component D α-subunit.

In the human gut microbiota, α-amino acids are used for energy production and as valuable sources of carbon and nitrogen. Members of the Clostridiales order are prolific amino acid metabolizers, using a specialized form of anaerobic metabolism known as Stickland fermentation (Fig. 1A) [21–23]. In this amino acid fermentation pathway, a redox reaction occurs between two amino acids to generate ATP. One amino acid, the electron donor, is oxidized to a fatty acid with one fewer carbon than the amino acid precursor. The second amino acid acts as the electron acceptor and is reduced to a fatty acid with the same carbon skeleton as the amino acid precursor. Twelve of the twenty proteinogenic amino acids are known to be metabolized via Stickland fermentation [24]. Whereas some amino acids act only as electron donors (e.g. serine, cysteine) or acceptors (e.g. glycine, proline), others can serve both roles (e.g. leucine, aromatic amino acids) [24–26].

The ability to generate ATP from amino acid metabolism provides a growth advantage for many commensal and pathogenic Clostridia in the human gut [25, 27]. Amino acid consumption by commensal strains serves to protect the host from the nosocomial pathogen *Clostridioides difficile* [28]. In the absence of other competing Stickland fermenters, *C. difficile* metabolizes available amino acids to establish colonization and infection [29, 30]. Additionally, amino acid fermentation has been correlated with toxin production [31–33]. Thus, modulation of the gut microbiota to enrich for Stickland fermenting organisms is a potential avenue to prevent and treat *C. difficile* infection [28].

Additionally, Stickland fermentation of aromatic amino acids generates many unique microbial metabolites connected to human health [34, 35]. Tryptophan metabolites promote intestinal epithelial barrier function by acting as ligands for host receptors. For example, indoleacrylic acid, indole-3-aldehyde, and indole-3-propionic acid activate the pregnane X receptor, a regulator of intestinal permeability via TLR4 [36, 37]. Indole-3-propionic acid and phenylpropionic acid derived from phenylalanine act as aryl hydrocarbon receptor ligands, causing downstream activation of IL-22 to promote innate immune defense and wound healing [34, 38, 39]. Consequently, lower levels of these metabolites are correlated with inflammatory bowel disease (IBD) [34, 37]. Tryptophan metabolites were also found to enhance barrier protection against pathogens and food antigens in patients with ulcerative colitis [38]. Likewise, tyrosine derivatives, such as 3-hydroxy-phenylpropionic acid (HPPA) and 4-hydroxy-phenylpropionic acid, have health-promoting effects. For example, HPPA supplementation in mice lowered the levels of lipids in serum and the liver and diminished phenotypes of non-alcoholic fatty liver disease [35]. These examples highlight the local and systemic effects on the host physiology of bacterial amino acid metabolism.

A key step in the reductive fermentation of aromatic and branched-chain amino acids is the dehydration of a 2-hydroxyacyl-CoA to yield an α,β-unsaturated thioester (i.e. 2-enoyl-CoA) (Fig. 1A) [24]. Reduction of the 2-enoyl-CoA product is one of the key steps that generates oxidized NAD^+^ equivalents in Stickland fermentation. While dehydration is typically a facile biochemical reaction, the elimination of a hydroxyl group at the alpha position of a carboxylic acid or thioester is chemically more challenging [23]. This reaction requires the removal of a relatively weakly acidic β-proton, which cannot be achieved with biological proteinogenic bases. Instead, this critical reaction in Stickland fermentation is catalyzed by a class of Fe–S cluster-dependent enzymes called 2-hydroxyacyl-CoA dehydratases (HADs) through an unusual radical-based mechanism [24].

HADs constitute a structurally and mechanistically unique family of Fe–S cluster enzymes. There are two major components required for dehydration: the dehydratase (i.e. component D; IPR010327) and the activase (i.e. component A; IPR008275) [24]. Component D functions as an αβ heterodimer of two structurally homologous proteins [40]. Each subunit harbors a [Fe_4_S_4_] cluster, positioned 12 Å from each other at the dimer interface (Fig. 1B) [40]. Some dehydratases (e.g. 2-hydroxyglutaryl-CoA dehydratase, HgdA) also harbor an organic flavin cofactor in addition to the Fe–S cluster [24]. The [Fe_4_S_4_] cluster of the α-subunit is located in the enzyme active site where dehydration occurs. In the crystal structure of 2-hydroxyisocaproyl-CoA dehydratase, only three of the four iron ions of the cluster are ligated by cysteine residues. The fourth iron ion has an open coordination site to engage in catalysis, which the authors modeled with the 2-hydroxyacyl-CoA substrate as a ligand [40]. This cluster is proposed to reduce the substrate, generating a critical radical intermediate as described below, and act as a Lewis acid for hydroxyl group elimination (Fig. 1C) [23, 41]. In the β-subunit, the [Fe_4_S_4_] cluster is also ligated by only three proteinaceous cysteines and a fourth unidentified thiol ligand [40]. The β-subunit cluster serves roles in electron transfer and storage during catalysis. Both Fe–S clusters of component D have very low reduction potentials and require a specialized activating partner protein for reduction [42]. The activase, or component A, is also a Fe–S cluster-dependent enzyme that has ATPase activity [24, 43, 44]. These unique proteins, known as “Archerases,” have a homodimeric structure with the [Fe_4_S_4_] cluster located at the dimer interface (Fig. 1B) [45]. This highly solvent exposed cluster is ligated by two cysteine residues from each monomer, creating a structural hinge that converts from an extended to a bent conformation upon ATP hydrolysis [45]. This ATP-induced conformational change drives electron transfer from the [Fe_4_S_4_] cluster of the activase to the β-subunit in component D [44]. This mode of reductive activation is analogous to the ATP-dependent activation of nitrogenase; however, the two activases do not share structural homology, suggesting convergent evolution of this function [44]. Altogether, multiple Fe–S proteins with distinct functions are required to enact the dehydration of 2-hydroxyacyl-CoA substrates.

The proposed mechanism of dehydration relies on one-electron reduction of the substrate via the protein Fe–S clusters (Fig. 1C) [41, 46]. The first step in catalysis is the ATP-driven electron transfer from the [Fe_4_S_4_] cluster of component A to the β-[Fe_4_S_4_] cluster of component D [44, 45]. This reducing equivalent is then transferred from the β-subunit to the adjacent [Fe_4_S_4_] cluster in the α-subunit [40]. The α-[Fe_4_S_4_] cluster then reduces the coordinating substrate by one electron to generate a ketyl radical anion [46]. This critical intermediate enables elimination of the hydroxyl group at C2, likely assisted by direct coordination to the α-[Fe_4_S_4_] cluster, and generates an enoxy radical species [46]. The enhanced acidity of the β-protons in this intermediate compared to the substrate enable deprotonation at C3, yielding an allylic ketyl radical anion species [47]. Finally, the 2-enoyl-CoA product is generated upon electron transfer to the α-[Fe_4_S_4_] cluster [46]. This reducing equivalent is subsequently transferred to the β-[Fe_4_S_4_] cluster where it can be used in another round of catalysis [23]. Thus, the heterodimer structure of component D functions to store the reducing equivalent, requiring only a single activation by component A for thousands of rounds of catalysis [48].

### Radical SAM GRE-activating enzymes in disease-associated metabolism

**Figure 2.**
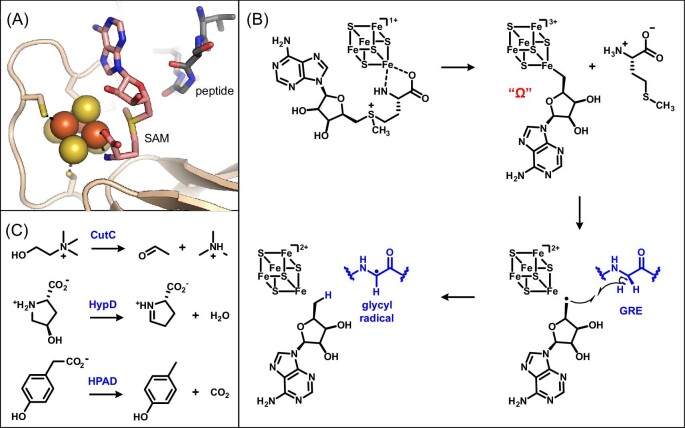
Radical SAM GRE activating enzymes involved in disease-associated chemistry. (A) Active site of pyruvate formate-lyase activating enzyme (PDB 3CB8) with SAM (pink sticks) bound to the Fe–S cluster and a peptide substrate mimic (grey sticks) bound in close proximity to the 5ʹ-carbon of SAM. (B) Current proposed mechanism of radical SAM enzymes, involving reductive cleavage of SAM to form the organometallic omega intermediate, followed by 5ʹ-deoxyadenosyl radical formation. GRE activation involves abstraction of the pro-*S* hydrogen atom from a glycine residue by the 5ʹ-deoxyadenosyl radical. (C) Key reactions in the human gut microbiota catalyzed by GREs, which require activation by radical SAM enzymes.

Enzymes belonging to the radical SAM superfamily (IPR007197) are prevalent in anaerobic bacteria to enact C-H activation chemistry [49–52]. Radical SAM enzymes possess a critical [Fe_4_S_4_] cluster that functions in substrate binding and reductive catalysis (Fig. 2A) [50, 53]. Three of the four iron ions in the cluster are ligated by cysteine residues found in a conserved CX_3_CX_2_C sequence motif [50, 53]. The fourth iron ion has an open coordination site in the resting state and then directly binds the co-substrate *S*-adenosyl-l-methionine (SAM) via the carboxy and amino groups during catalysis (Fig. 2A) [50, 53]. Upon SAM binding and one-electron reduction of the [Fe_4_S_4_]^2+^ cluster, the enzyme catalyzes reductive cleavage of SAM to generate l-methionine and a 5ʹ-deoxyadenosyl radical [54–56]. More recent evidence demonstrates that this short-lived radical forms an organometallic intermediate with a bond between the 5ʹ carbon and the unique iron of the Fe–S cluster (Fig. 2B), known as the “omega state” [56–58]. Homolysis of this bond initiates catalysis by regenerating the 5ʹ-deoxyadenosyl radical to serve as a potent oxidant for C–H activation chemistry at the enzyme active site (Fig 2B) [56]. Radical SAM enzymes have diverse functions in human gut microbes, including natural product biosynthesis, post-translational protein modifications, and vitamin biosynthesis [14].

Glycyl radical enzyme (GRE) activating enzymes (GRE-AEs) are a particularly important subclass of the radical SAM superfamily for microbial metabolism in the human gut [14, 49, 50, 59]. GRE-AEs use the 5ʹ-deoxyadenosyl radical intermediate to modify the peptide backbone of a partner GRE, installing a key catalytic glycyl radical (Fig. 2B) [59]. Two well-studied GREs, class III ribonucleotide reductase (RNR) and pyruvate formate-lyase (PFL), perform essential biochemical reactions in obligate and facultative anaerobes. RNR produces the 5ʹ-deoxyribonucleotide monomers of DNA, and PFL generates acetyl-CoA used for ATP production in anaerobic glucose metabolism [60, 61]. Whereas these two GREs are quite ubiquitous across anaerobic organisms, other GREs perform more specialized biochemical reactions in select gut bacteria (Fig. 2C) [14]. Two functional categories of GREs are involved in microbial metabolism in the human gut: those performing elimination reactions (i.e. eliminases) and those catalyzing decarboxylation reactions (i.e. decarboxylases) [62]. Many of these GREs catalyze biochemical reactions with important disease implications for commensal and pathogenic bacteria [14].

The enzyme choline TMA-lyase (CutC) is a GRE found in commensal gut bacteria that has strong links to human disease [14]. CutC and its activating enzyme, CutD, convert dietary choline to acetaldehyde and trimethylamine (TMA) (Fig. 2C) [63, 64]. The latter product is directly associated with the genetic disorder trimethylaminuria [65]. Under normal conditions, TMA is absorbed from the colon, oxidized in the liver, and excreted in urine. However, genetic mutations in the host liver monooxygenase cause accumulation of TMA in the body and a fishy malodor characteristic of the genetic disorder trimethylaminuria [66]. The oxidized product of TMA, trimethylamine-*N*-oxide (TMAO), is also associated with many other systemic human diseases. Studies in animal models and humans indicate that TMAO itself and TMA-producing microbes have causal roles in the development of cardiovascular disease [67]. High levels of TMAO are also correlated with increased risk for metabolic diseases, kidney disease, and non-alcoholic fatty liver disease [68, 69]. Thus, dietary or microbiota-based interventions that modulate TMA production are attractive therapeutic strategies to mitigate disease. Organisms that encode for the choline TMA-lyase CutC are typical members of a healthy human gut microbiota [64]; however, with high intake of choline from the diet, they produce a metabolite that is detrimental to human health. This example of host-microbial metabolism highlights an important connection between diet, the commensal microbiota, and human disease.

Two GREs, *trans*-4-hydroxy-l-proline dehydratase (HypD) and 4-hydroxyphenylacetate decarboxylase (HPAD), confer unique metabolic capabilities to the intestinal pathogen *C. difficile. Trans*-4-hydroxy-l-proline is a non-canonical amino acid found in mammalian and plant proteins, such as the structural protein collagen. The GRE eliminase HypD and its radical SAM activating enzyme are responsible for the dehydration of *trans*-4-hydroxy-l-proline to 1-pyrroline-5-carboxylate in Firmicutes and Bacteroides bacteria (Fig. 2C) [70, 71]. Clostridia, including *C. difficile*, use this reaction for energy production by reducing the HypD product to l-proline, which can then serve as an electron acceptor in Stickland fermentation [72, 73]. Studies in mouse models suggest that metabolism of *trans*-4-hydroxy-l-proline and other amino acids may play a key role in *C. difficile* infection and disease progression. An unbiased metabolomics study also showed that *trans*-4-hydroxy-l-proline is one of the primary metabolites rapidly consumed during early stages of infection [29]. Additionally, major shifts in amino acid metabolism are observed during inflammation, and coincide with collagen degradation, a key source of proline and *trans*-4-hydroxy-l-proline [30]. Lastly, using a *hypD* knockout strain of *C. difficile*, a modest reduction in fitness and toxin production was observed [73]. This organism's pathogenicity is also promoted by the unique ability to produce *p*-cresol via the GRE decarboxylase HPAD. This enzyme catalyzes decarboxylation of the tyrosine-derived metabolite 4-hydroxyphenylacetate (Fig. 2C), yielding the toxic metabolite *p*-cresol [74]. Despite its toxicity for most bacteria, *C. difficile* can survive in high concentrations of this phenolic molecule [75]. Notably, hypervirulent strains are correlated with higher levels of *p*-cresol production and higher tolerance to *p*-cresol [76]. Thus, these two examples of GRE-mediated metabolism confer a competitive advantage to this important pathogen in the gut microbiota.

### Flavin-dependent reductases in metabolism of endogenous and dietary molecules

**Figure 3.**
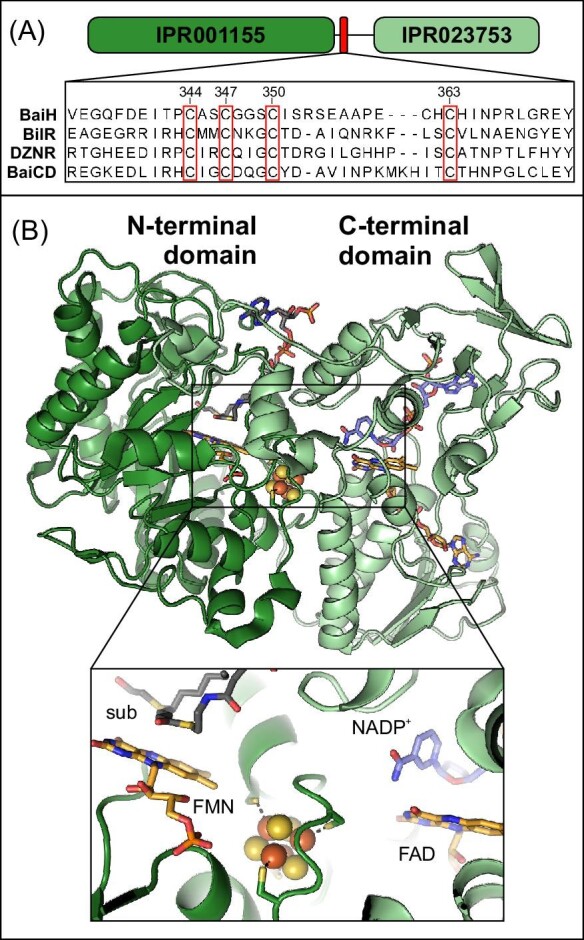
Two-domain architecture of Fe–S cluster-dependent flavoenzymes. (A) Cartoon depiction of the Fe–S flavoenzyme protein sequence, highlighting regions of domain homology and the location of the conserved cysteine motif. A segment of a multiple sequence alignment of gut microbial Fe–S flavoenzymes (UniProt IDs: P32370, A0A829NF98, M9NZ71, P19410) is shown with residue numbering corresponding to the BaiH protein sequence. (B) X-ray crystal structure of archetypal family member 2,4-dienoyl-CoA reductase from *E. coli* (PDB 1PS9). Organic cofactors and substrate analog are shown as sticks and the [Fe_4_S_4_] cluster is shown as spheres.

Fe–S cluster-dependent flavoenzymes are a class of oxidoreductases involved in gut microbial metabolism of endogenous and dietary molecules. These proteins are composed of two structural domains that harbor multiple organic cofactors and an iron-sulfur cluster (Fig. 3). The N-terminal NADH/flavin oxidoreductase domain (IPR001155) has a TIM-barrel fold and is characteristic of the FMN-binding “Old Yellow Enzyme” (OYE) family [77]. The C-terminal FAD/NAD(P)-binding domain (IPR023753) shares homology with the family of pyridine nucleotide-disulfide oxidoreductases and has binding sites for FAD and NAD(P)^+^ cofactors. In most cases, these two domains are fused as a single polypeptide; however, there are rarer instances where they are encoded as two separate proteins [78]. The intervening sequence between these two domains contains a conserved CX_2_CX_3_CX_11-13_C sequence motif (Fig. 3A). Based on structural studies of the family member 2,4-dienoyl-CoA reductase from *Escherichia coli*, this motif is predicted to bind a cubane [Fe_4_S_4_] cluster positioned at the interface of the N- and C-terminal domains as part of an electron transport chain (Fig. 3B) [79]. The Fe–S cluster mediates electron transfer from the reduced FAD cofactor in the C-terminal domain to the FMN cofactor in the active site of the N-terminal domain, ultimately shuttling two reducing equivalents from NAD(P)H to the substrate.

**Figure 4.**
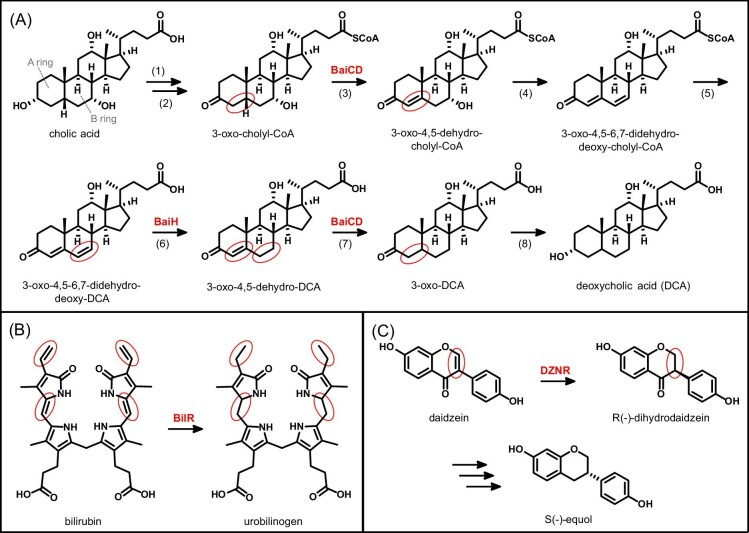
Reduction reactions catalyzed by Fe–S cluster-dependent flavoenzymes in the gut microbiota. (A) Biochemical pathway for 7-α-dehydroxylation of primary bile acids. (B) Reduction of heme-derived bilirubin to urobilinogen. (C) Metabolism of soy isoflavone daidzein to (*S*)-equol.

In the human gut microbiota, members of this enzyme family are known to participate in the reductive metabolism of bile acids, heme, and flavonoids (Fig. 4). As described in more detail below, the microbial modifications to these complex organic substrates significantly impact their bioavailability and bioactivity in the human body. However, the vast majority of Fe–S flavoenzymes have unknown functions [80]. This family is especially prevalent in Clostridia; approximately 70% of Clostridial genomes encode at least one enzyme from this family and some strains encode >10 different homologs [80]. This evolutionary diversification suggests that their role in Clostridial metabolism and the gut microbiota is only beginning to be revealed.

Fe–S flavoenzymes catalyze multiple reactions in the reductive metabolism of primary bile acids. These modified steroids derived from cholesterol are secreted into the large intestine to aid in digestion and absorption of fats and vitamins. However, gut bacteria extensively modify them through deconjugation and reductive dehydroxylation, generating a large pool of secondary bile acids [81, 82]. These microbial products are absorbed into the bloodstream and exert unique effects on host biology. For example, secondary bile acids act as ligands for human receptor proteins, such as G protein-coupled bile acid receptor 1 (TGR5) and nuclear hormone receptor farnesoid X receptor (FXR) [83]. Importantly, these receptor ligand functions are associated with host metabolic diseases (e.g. obesity and diabetes), inflammatory disease, and colon cancer [83].

Members of the Clostridium cluster XIVa are primarily responsible for converting the primary bile acids cholic acid and chenodeoxycholic acid to deoxycholic acid (DCA) and lithocholic acid, respectively, via 7-α-dehydroxylation (Fig. 4A) [84, 85]. In this pathway, the A and B rings of the steroid core are initially oxidized to enable 7-α-hydroxyl group elimination, followed by their reduction to regenerate the steroid core (Fig. 4A) [85]. This metabolism is encoded by the bile-acid-inducible (*bai*) operon, which includes two Fe–S flavoenzyme homologs, BaiH and BaiCD [84, 85]. The enzyme BaiH is responsible for reducing the B ring of 3-oxo-4,5-6,7-didehydro-DCA to 3-oxo-4,5-dehydro-DCA (step 6). The enzyme BaiCD is involved in two steps of the pathway, one oxidative step (step 3) converting 3-oxo-cholyl-CoA to 2-oxo-4,5-dehydrocholyl-CoA and one reductive step (step 7) converting 3-oxo-4,5-dehydro-DCA to 3-oxo-DCA [85]. A similar reduction of the A ring occurs in the microbial metabolism of cholesterol to coprostanol in the gut [86]; however, the reductase performing this reaction has not yet been identified. Thus, Fe–S flavoenzymes may be involved more broadly in steroid metabolism by the human gut microbiota.

A Fe–S flavoenzyme was also recently identified to participate in host-microbe co-metabolism of heme. Bilirubin is the major product of host heme degradation, and is secreted as a sugar conjugate via bile into the large intestine for excretion [78]. Gut bacteria deconjugate the diglucuronide form of bilirubin in the colon, allowing it to be reabsorbed into the host enterohepatic circulation. Alternatively, microbes can convert unconjugated bilirubin to more readily excreted metabolites, such as urobilinogen and stercobilinogen [78]. Therefore, circulating bilirubin levels can either increase or decrease, depending on the specific metabolic activities encoded in the microbiome. At low levels, the antioxidant properties of bilirubin confer cytoprotective effects; however, bilirubin is cytotoxic at high levels and causes jaundice and kernicterus [87]. Additionally, the microbial metabolite urobilinogen may be associated with incident heart failure and insulin resistance [88, 89].

The conversion of bilirubin to urobilinogen involves reduction of four alkene bonds (Fig. 4B) and is catalyzed by a single enzyme, bilirubin reductase (BilR) [78]. This Fe–S flavoenzyme has been identified in strains of *C. difficile, Thomasclavelia ramosa* (formerly *Clostridium ramosum*), *Clostridium perfringens*, and *Bacteroides fragilis* [78]. Although it is nearly universally present in healthy adults, the *bilR* gene was detected less frequently in the gut microbiome of patients with IBD, as well as infants with high neonatal jaundice risk [78]. The discovery of this enzyme will enable further studies to understand the impact of the microbiota on heme processing and excretion. Additionally, future biochemical studies will provide insight into how this enzyme targets two different functional groups of its substrate.

In addition to endogenous molecules, Fe–S flavoenzymes also modify compounds from the diet. Plant flavonoids are a large class of bioactive polyphenols that are metabolized by the human gut microbiota [90, 91]. For example, gut bacteria convert the soybean isoflavone daidzein to the health-associated metabolite (*S*)-equol (Fig. 4C). This microbial metabolite is an estrogen receptor β agonist and associated with positive outcomes for vasomotor symptoms, osteoporosis, prostate cancer, and cardiovascular risk [92]. Metabolism of daidzein is restricted to only a handful of gut bacteria, including *Slackia isoflavoniconvertens, Slackia sp.* strain NATTS, *Lactococcus sp.* strain 20-92, and *Eggerthella sp*. strain YY7918 [93, 94]. These organisms encode for a pathway involving three consecutive reduction steps to produce (*S*)-equol. The Fe–S flavoenzyme daidzein reductase (DZNR) performs the first step in the pathway, reducing the double bond in the C ring to produce dihydrodaidzein (Fig. 4C) [93]. DZNR is also active toward the structurally related isoflavone genistein, which is metabolized to 5-hydroxy-equol [93]. In addition to isoflavones, other structural classes of flavonoids (e.g. flavonols and flavones) contain an alkene in the C ring, which could be substrates for DZNR homologs. Thus, Fe–S flavoenzymes may be involved in microbial metabolism of other bioactive dietary flavonoid molecules.

### U32 oxidases in adaptation to hypoxic environments

While reductive chemistry dominates in anoxic environments such as the human gut, oxidative reactions are also necessary for critical biochemical processes. Oxidases typically use molecular oxygen as an electron acceptor in catalysis or directly as a co-substrate. Conversely, distinct enzymatic strategies are required for oxidative chemistry in the absence of oxygen. Oxygen-insertion reactions in particular require alternative mechanisms. An intriguing new class of Fe–S cluster-dependent oxidases, herein called “U32 oxidases,” was recently identified to perform O_2_-independent hydroxylation reactions in bacteria [95–98]. While characterized representatives come from the model organisms *E. coli* and *B. subtilis*, members of this family are widespread throughout the bacterial kingdom.

**Figure 5.**
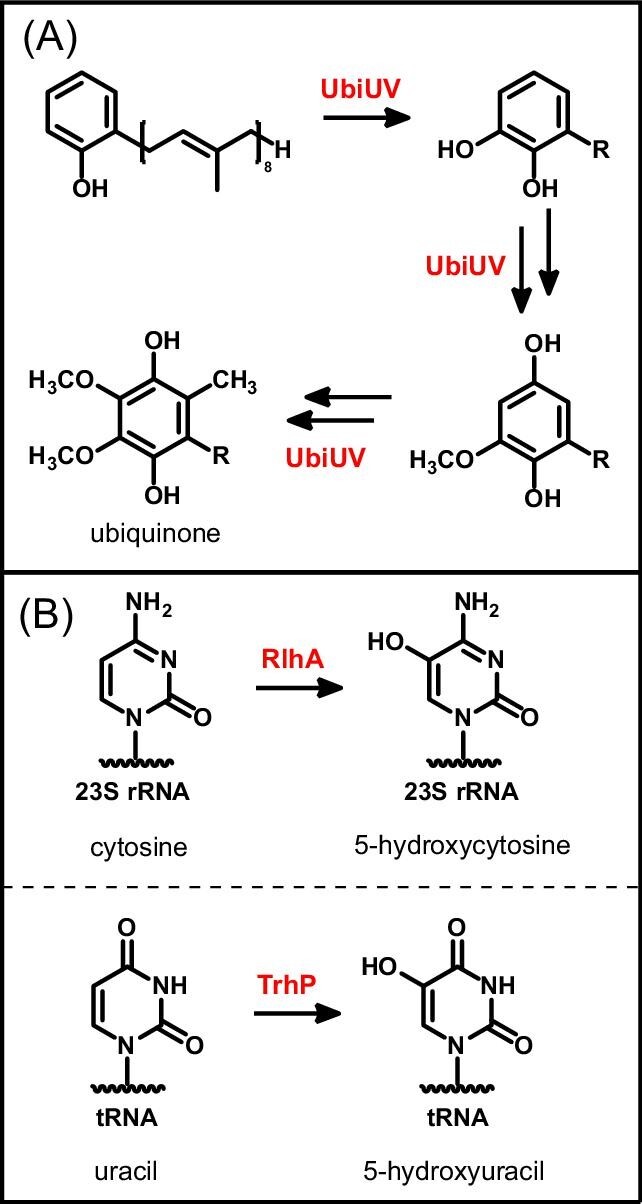
O_2_-independent hydroxylation reactions catalyzed by Fe–S cluster-dependent U32 oxidases. (A) Proposed hydroxylation reactions performed by the pair of U32 oxidases UbiU and UbiV in anaerobic ubiquinone biosynthesis. (B) rRNA and tRNA nucleotide modifications installed by U32 oxidases.

Biochemically characterized U32 oxidases participate in anaerobic ubiquinone biosynthesis and RNA base modification (Fig. 5). In bacteria such as *E. coli*, ubiquinone is an essential electron carrier for aerobic respiration [99]. However, this quinone may also function in anaerobic conditions, enabling bacterial adaptation to oxygen availability [100]. For example, strains of *Pseudomonas aeruginosa* with mutations of the anaerobic ubiquinone U32 oxidases *ubiU* and *ubiV* genes demonstrated growth defects when relying on anaerobic denitrification for energy production [101]. Additionally, an *E. coli* knockout strain of the *ubiU* and *ubiV* genes showed a modest decrease in fitness when colonizing a mouse gut compared to the wild-type strain [100]. U32 oxidases that install RNA modifications may also play a role in bacterial adaptation to hypoxia. In a model organism of *Mycobacterium*, tRNA modifications involving base hydroxylation were correlated with oxygen availability when cultured in hypoxic conditions [102]. Considering the microoxic to anoxic conditions of the human gut, U32 oxidases may contribute to survival and adaptation of commensal organisms and pathobionts in this habitat.

U32 oxidases constitute a new structural family of Fe–S proteins. They belong to the largely uncharacterized family of “U32 peptidase” proteins (IPR001539) and are predicted to share a (βα)_8_ TIM-barrel fold, based on the experimental structure of one uncharacterized member (PDB 5D88) [103]. Although radical SAM enzymes also adopt this fold, U32 proteins lack the canonical cysteine sequence motif that is highly conserved in the radical SAM superfamily. Instead, U32 oxidases typically have four conserved cysteines in a CX_6_CX_15_CX_3_C sequence motif that is essential for catalytic activity [95–98]. Spectroscopic studies of the ubiquinone U32 oxidases suggest that these cysteines coordinate a [Fe_4_S_4_] cluster [95]; however, structural studies are lacking to provide additional insight into the metallocofactor structure and coordinating ligands. Although the U32 domain is predicted to have only one cofactor binding site, U32 oxidases often function as oligomers, similar to the HAD proteins described above. For example, the ubiquinone biosynthesis proteins UbiU and UbiV form a heterodimer [95], despite sharing low sequence identity. Similarly, the low-identity homologs TrhP1 and TrhP2 (formerly known as YrrN and YrrO) from *Bacillus subtilis* form a heterodimer [96]. The RlhA protein consists of only one U32 domain but appears to have an extra domain of unknown function with highly conserved cysteine residues capable of binding a metallocofactor, which may obviate the need for dimerization [97]. The complex oligomeric and multi-domain architecture of U32 oxidases suggests that more than one Fe–S cluster is necessary for catalysis, consistent with the two-electron oxidation chemistry performed by these enzymes.

The mechanism of hydroxylation used by U32 oxidases is anticipated to invoke unique reactivity for Fe–S cluster enzymes. Other well-known O_2_-independent hydroxylases are molybdenum-dependent enzymes, which use water as the source of oxygen [104]. In these enzymes, the molybdenum center activates the substrate or water in a nucleophilic attack mechanism to form the new carbon-oxygen bond [104]. Other O_2_-independent hydroxylases belong to the radical SAM enzyme family and similarly use water as the oxygen donor [105]. In contrast, the hydroxyl group in anaerobic ubiquinone biosynthesis derives from a unique organic source, the shikimate-derived molecule prephenate [106]. Genetic mutations in prephenate also prevent RNA modifications installed by U32 oxidases, suggesting that this oxygen source may be conserved across the U32 oxidases [96–98]. However, the exact chemical transformation of prephenate and direct evidence for U32 oxidases catalyzing this reaction remain to be demonstrated. In addition, the role(s) of the Fe–S clusters in catalysis have not yet been determined. While the Fe–S clusters are likely to participate in electron transfer, they may also act in substrate activation. The Fe–S cluster-dependent enzyme IspH could provide insight into a possible catalytic role for the Fe–S cluster in substrate hydroxylation. This bacterial enzyme catalyzes reductive dehydroxylation of its substrate in isoprenoid biosynthesis and has been extensively studied due to its promise as a therapeutic target [107]. The current proposed mechanism for IspH invokes direct coordination of the substrate to the Fe–S cluster and involvement of an organometallic intermediate during dehydroxylation [108, 109]. A similar mechanism could be envisioned for U32 oxidases; however, many open questions remain about the structural and electronic properties that promote oxidative catalysis and C–O bond formation required for substrate hydroxylation.

### Molybdenum-dependent enzymes

**Figure 6.**
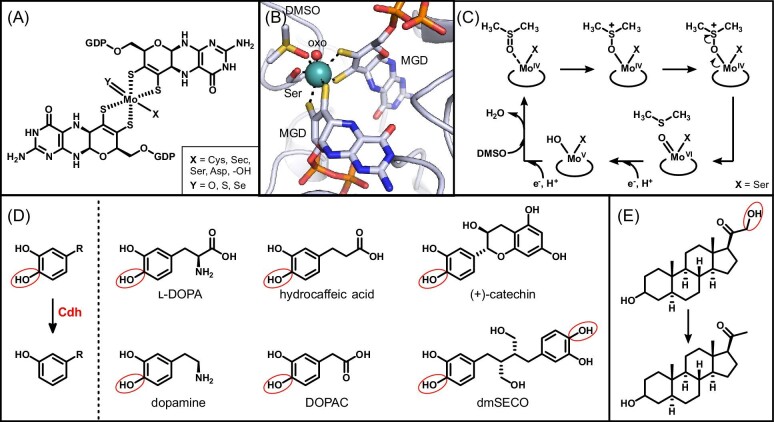
Molybdenum-dependent enzymes of the DMSO reductase family catalyzing oxygen atom transfer reactions using inorganic and organic substrates. (A) Structure of the bis-MGD molybdenum cofactor common to the DMSO reductase family. (B) Active site depiction of DMSO reductase (PDB 4DMR) showing the molybdenum bis-MGD cofactor with a terminal oxo ligand (red sphere), coordinating serine residue, and the DMSO substrate bound to the molybdenum ion (teal sphere). (C) Proposed reaction mechanism for DMSO reductase. (D) Known substrates of molybdenum-dependent catechol dehydroxylase (Cdh) enzymes. (E) Corticoid dehydroxylation catalyzed by a molybdenum-dependent enzyme from *E. lenta*.

Molybdenum cofactors are versatile protein prosthetic groups utilized in anaerobic microbial chemistry [104, 110]. These bioinorganic cofactors have a central molybdenum ion coordinated by a dithiolate group of one or two molybdopterin ligands and variable inorganic and proteinaceous amino acid ligands (Fig. 6A) [104, 110]. The nature of the ligands and geometry of the cofactor significantly impact the electronic properties and reactivity of the molybdenum center, enabling a wide range of chemical transformations. In this section, we focus on molybdenum enzymes belonging to the dimethyl sulfoxide (DMSO) reductase family (IPR006657), which use a bis-molybdopterin guanidine dinucleotide (bis-MGD) molybdenum cofactor (Fig. 6A,B). We describe two functional classes of this family and their roles in reductive chemistry in the human gut microbiota.

### Reductases in anaerobic respiration of inorganic substrates

Molybdenum-dependent enzymes are a major class of respiratory reductases in the human gut microbiota [111, 112]. They enable the use of a wide range of hosts, diet, and other xenobiotic molecules as alternative terminal electron acceptors for anaerobic respiration [112]. Well known examples reduce inorganic substrates, including DMSO reductase, nitrate reductase, and sulfate reductase. However, as described in the next section, respiratory reductases using organic substrates also play important roles in the human gut. Many gut microbes encode for multiple molybdenum-dependent respiratory reductases, conferring the metabolic versatility to adapt to available nutrients and environmental conditions [112].

The ability to generate energy from anaerobic respiration is a beneficial alternative to anaerobic fermentation for many commensal and pathogenic bacteria. For example, the human gut pathogen *Salmonella enterica* uses a specific molybdenum-dependent respiratory reductase to promote growth in inflammatory conditions [113]. During infection, *Salmonella* induces a host inflammatory response, which involves production of reactive oxygen species. This stress response results in the production of oxidized molecules in the gut, such as the thiosulfate tetrathionate [113]. *Salmonella* encodes for a molybdenum enzyme, tetrathionate reductase, that catalyzes two-electron reduction of the disulfide bond in tetrathionate [113, 114]. This enzymatic activity enables *Salmonella* to use this unique terminal electron acceptor, conferring a competitive advantage over commensal gut microbes in a mouse model of inflammation [113].

The respiratory reductases belonging to the DMSO reductase family share a common fold for binding the molybdenum cofactor, but they have a wide variety of structural architectures and accessory redox cofactors [110, 111, 115, 116]. The minimal structure is composed of four domains and harbors the catalytic active site with the molybdenum bis-MGD cofactor [110, 111, 115]. This cofactor has a central molybdenum ion coordinated by two pyranopterin molecules, a terminal oxo ligand, and a variable amino acid residue (i.e. serine, cysteine, selenocysteine, aspartate) or a terminal hydroxide ligand (Fig. 6A,B). In some cases, the catalytic domain also harbors a [Fe_4_S_4_] cluster in close proximity to the molybdenum cofactor [110]. More structurally complex members of this family form oligomers with additional protein subunits that contain accessory Fe–S clusters or heme cofactors for electron transfer [110]. Additionally, while some respiratory reductases are cytosolic, others possess membrane anchoring subunits that localize the protein complex to the periplasm [110, 111].

The molybdenum bis-MGD cofactor of these enzymes mediates two-electron reduction reactions, resulting in removal of an oxygen atom from the inorganic substrate or cleavage of a heteroatom-heteroatom bond. The current proposed mechanism for this family is based on the archetypal member DMSO reductase (Fig. 6C) [115, 116]. In the resting state, the cofactor has an Mo(VI) ion with a terminal oxo ligand. An electron and proton transfer event reduces the Mo(VI) center to Mo(V) and converts the oxo ligand to a hydroxide ligand. A second reduction event converts the hydroxide ligand to a labile water ligand and generates a five-coordinate Mo(IV) intermediate. Direct coordination of the substrate to the Mo(IV) ion then initiates oxygen atom transfer. This step is proposed to occur via a concerted two-electron transfer from the molybdenum center to the substrate, regenerating the oxidized Mo(VI) resting state with a terminal oxo ligand originating from the substrate.

### Dehydroxylases in metabolism of xenobiotic and endogenous organic molecules

A subset of molybdenum-dependent reductases specialize in the metabolism of catechol-containing molecules originating from the host, diet, and drugs [112, 116]. These enzymes catalyze catechol dehydroxylation at the *para* position of the substrate, resulting in a 3-hydroxy-substituted phenol product (Fig. 6D) [116]. Catechol dehydroxylases are predominantly found in two related genera of host-associated Actinomycetota—*Eggerthella* and *Gordonibacter* [112, 117]. These organisms often encode for multiple dehydroxylase homologs, highlighting significant evolutionary expansion of these enzymes within these genera [112, 117]. Characterized members of this family demonstrate high specificity for their substrates, which in turn tightly regulate enzyme expression [112, 117]. These catechols are reduced as alternative electron acceptors for anaerobic respiration in *Eggerthella* and *Gordonibacter*, conferring a growth advantage to these organisms in the competitive gut microbiota ecosystem [117].

The host neurotransmitter dopamine is used as the substrate of a molybdenum-dependent dehydroxylase [117, 118]. This catecholamine is abundant in the gastrointestinal tract, constituting ∼50% of total endogenous dopamine [119], and its depletion may be implicated in gut motility and pathogen colonization [120, 121], Strains of *Eggerthella lenta* that encode for dopamine dehydroxylase (Dadh) convert dopamine to the derivative *m*-tyramine (Fig. 6D) [117, 118]. This reaction is also part of a two-step pathway for microbial degradation of the Parkinson's drug levodopa (l-dopa) in the gut (Fig. 6D) [118]. This microbial metabolism inactivates l-dopa, contributing to the heterogeneity observed in patient response to this medication [118]. Similar catechol moieties are present in other pharmaceutical drugs and may be subject to microbial metabolism via molybdenum-dependent dehydroxylases [116].

Substrate-specific catechol dehydroxylases have also been linked to gut microbial metabolism of the dopamine derivative 3,4-dihydroxyphenylacetic acid (DOPAC) and the dietary catechols (+)-catechin, hydrocaffeic acid, and didemethylsecoisolariciresinol (dmSECO) (Fig. 6D) [117, 122]. These dehydroxylases all belong to the DMSO reductase family of molybdenum-dependent enzymes; however, they have distinct protein architectures and cofactors depending on their native organism [116]. The catechin and hydrocaffeic acid dehydroxylases from *Eggerthella* species have three protein components—the catalytic bis-MGD subunit (α), an electron transport subunit predicted to harbor four Fe–S clusters (β), and a membrane anchor subunit (γ) [116]. On the other hand, the DOPAC and dmSECO dehydroxylases from *Gordonibacter* species have a simpler two-component αβ architecture [116]. They are also predicted to use fewer Fe–S cluster cofactors than the *Eggerthella* systems [116]. Notably, the catalytic subunits of the *Eggerthella* dehydroxylases possess a Fe–S cluster binding motif that is absent in the *Gordonibacter* proteins [116]. Despite these structural differences, catechol dehydroxylases form a distinct phylogenetic clade within the DMSO reductase family [117]. Bioinformatic analyses suggest that many more catechol dehydroxylase homologs exist, and given their recent discovery in the last five years, many novel biochemical functions are likely to be uncovered within this family [112, 116, 117].

Until recently, molybdenum-dependent dehydroxylases were known to act exclusively on catechol substrates. Intriguingly, a novel molybdenum-dependent reductase from gut *Eggerthella* and *Gordonibacter* species was discovered to catalyze dehydroxylation of host corticoids [123]. These molecules lack a catechol moiety, and instead, the hydroxyl group removed during catalysis is located at the alpha position of an external ketone on the steroid core (Fig. 6E). This discovery opens the possibility that molybdenum-dependent enzymes catalyze dehydroxylation reactions on a wider range of substrates than previously appreciated. This newly identified reaction also has important implications for human health. Corticoids are human steroids with immune and metabolic functions [123]. Microbial dehydroxylation converts corticoids into a distinct class of molecules called progestins, which act as sex hormones and neurosteroids [123]. Thus, this microbial modification drastically changes the bioactivity of these steroids in the human body.

## Opportunities for metalloenzyme discovery in the human gut microbiota

### Bioinformatic analysis of metalloenzyme families in human gut microbes

**Figure 7.**
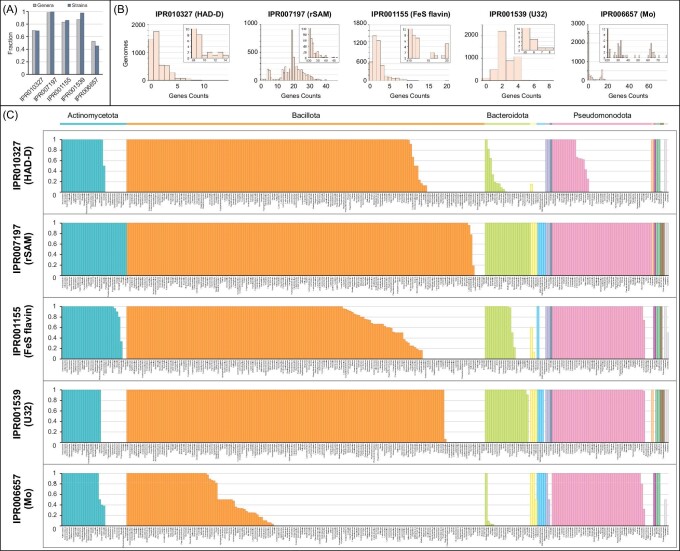
Bioinformatic analysis of metalloenzyme family representation in human gut microbes. (A) Fraction of all genera or strains encoding at least one member of the metalloenzyme family. (B) Histogram plots for each metalloenzyme family of gene counts per genome. (C) Fraction of each genus encoding at least one member from the metalloenzyme family. Phyla from left to right: Actinomycetota (blue), Bacillota (orange), Bacteroidota (lime green), Campylobacterota (yellow), Euryarchaeota (cyan), Fusobacteriota (purple), Lentisphaerota (dark blue), Pseudomonadota (pink), Spirochaetota (light orange), Synergistota (magenta), Thermodesulfobacteriota (green), Verrucomicrobiota (brown), unclassified (grey).

Sequencing efforts have yielded a wealth of human gut microbial genomes and metagenomes to mine for novel metalloenzymes with unique biochemical reactions. To assess the prevalence of the metalloenzyme families highlighted in this review, we searched all available genomes of human gut microbes (∼5000 strains) deposited in the DOE Joint Genome Institute Integrated Microbial Genomes and Metagenomes database. Each metalloenzyme family is represented in >50% of the genera in this dataset (Fig. 7A), which includes 277 distinct bacterial genera from 11 phyla and 4 archaeal genera. This widespread distribution emphasizes the significance of these metalloenzymes and their functions for human gut microbes.

The radical SAM enzyme superfamily is quite ubiquitous across human gut microbes. At least one family member is found in >99% of all strains and 98% of all genera (Fig. 7A). Most strains also encode for multiple members of the radical SAM enzyme family, often ranging from 10 to 30 homologs per genome (Fig. 7B). While all members of the radical SAM enzyme superfamily were evaluated in this analysis, other published analyses have focused specifically on the subfamily of GRE-AEs and their corresponding GRE partners [70, 124]. Their results demonstrated high frequency of this enzyme class in the human gut microbiota and also revealed that many abundant GREs have unknown functions, presenting opportunities to discover new reactions of functional importance in the gut microbiota.

The distribution of the other metalloenzyme families is more variable within the major phyla of gut microbes (Fig. 7C). For example, HAD enzymes are present in only one third of genera in the Pseuodomonodota phylum and two thirds of the Actinomycetota phylum. The U32 enzymes are absent in one third of the genera in the Actinomycetota phylum and ∼10% of the Bacillota phylum (Fig. 7C). However, it should be noted that this analysis includes both Fe–S cluster-dependent oxidases and putative peptidases of the U32 enzyme family. A better understanding of sequence motifs to discern the two functional subclasses may reveal distinct distribution in gut microbes.

Beyond the phylum level, the distribution of metalloenzyme families can also be highly variable at the genus level. For instance, the Fe–S flavoenzymes exhibit significant strain variation within individual genera of the Bacillota phylum (Fig. 7C). Whereas some strains of the same genus do not encode for a single Fe–S flavoenzyme, others encode up to 20 homologs (Fig. 7B), as has been previously reported [80]. Similarly, the HAD enzyme family distribution is highly variable in the Actinomycetota, Bacillota, and Pseudomonodota phyla and most significantly in the Bacteroidota phylum (Fig. 7C). This strain-level variation suggests that these metalloenzymes may confer unique functions in select strains. More broadly, this analysis highlights the importance of strain-specific analyses when assessing metabolic functions in the human gut microbiota.

Compared to the Fe–S cluster-dependent enzyme families, the molybdenum-dependent enzyme family is found in relatively fewer gut microbes. It is represented in ∼50% of the genera and 45% of the strains analyzed (Fig. 7A). These enzymes are highly conserved in the Pseudomonodota phylum, but variably present in the Bacillota phylum and largely absent from the Bacteroidetes phylum (Fig. 7C). Although molybdenum-dependent dehydroxylases were not segregated in this analysis, they are known to occur in only two genera of Actinomycetota—*Eggerthella* and *Gordonibacter*. Consistent with previous reports [112, 117], our analysis shows that an astounding number of homologs (i.e. 30–60 different proteins) are encoded in individual strains of these bacteria (Fig. 7B). The diversification of this enzyme family may hint at its functional importance for the lifestyle of these microbes in the gut environment.

Overall, this bioinformatic analysis demonstrates that these classes of metalloenzymes are prevalent in human gut microbes, but the phylogenetic distribution is unique to each family. Additionally, the high copy number of homologs within individual organisms suggests that they contribute to the vast metabolic diversity of the microbiota. Lastly, the sheer number of family homologs reveals a substantial opportunity to understand the functions and roles of metalloenzymes in human gut microbes.

### Computational and experimental strategies for metalloenzyme discovery in the microbiota

The vast sequence and functional diversity of these metalloenzyme families offers exciting opportunities to discover new chemistry in the human gut microbiota. Each family described herein includes tens to hundreds of thousands of members; yet less than 1% of members have known functions. A combination of computational and experimental strategies is necessary to tackle the immense challenge of ascribing biochemical functions to microbial metalloenzymes.

The development of bioinformatic resources, such as the Enzyme Function Initiative (EFI) webtools [124, 125], has improved and accelerated our ability to analyze large protein families. Whereas phylogenetics is useful to analyze sequences on the scale of hundreds of proteins, sequence similarity networks (SSNs) enable analysis of millions of proteins. SSNs effectively reduce large superfamilies into a more manageable number of functional subgroups using only pairwise sequence alignments [124, 125]. This tool has been used to analyze numerous metalloprotein families, including the large radical SAM enzyme superfamily [126]. Sequence-based subfamily delineation could be further augmented with structural information using protein prediction programs, such as AlphaFold [127, 128]. However, further development of these tools is needed for the prediction of metallocofactor binding sites. A limited set of cofactors can be modeled using AlphaFold3 [129]; however, iron-sulfur clusters and molybdopterin cofactors are not included. While the AlphaFill module can often reliably model common organic cofactors [130], its utility is limited for inorganic cofactors. Considering that metallocofactors can dramatically impact tertiary structure, these developments are critical for the application of structure prediction programs to metalloproteins.

Beyond the structural folds and domains known to harbor metallocofactors, human gut microbes may also encode for unknown families of metalloproteins. A large proportion of proteins (∼40%) encoded by gut microbes cannot be assigned functions based on homology alone [13]. Structural prediction programs and machine learning approaches have the potential to identify domains of unknown function with conserved metal-binding residues (e.g. cysteine, histidine) clustered together in 3D space [131, 132]. Alternatively, experimental proteomic methods have shown promise for identifying metalloproteins. A chemoproteomics platform combined with iron-depletion growth conditions was recently used to profile metalloproteins in *E. coli* using mass spectrometry [133]. Notably, this method also directly detects coordinating cysteine residues because they are chemically modified in the procedure. A similar concept was applied to identify metalloproteins in mammalian cells, which could be readily adopted for bacteria. Instead of chemical modification, this method used thermal stability combined with metal chelation to identify metalloproteins by mass spectrometry [134]. These workflows could be used to profile known and unknown metalloproteins from human gut microbes.

Following their identification, the next problem to address is how to prioritize metalloproteins for functional characterization. This prioritization problem can be viewed from a microbe-centric or host-centric perspective. Taxonomic distribution can be used to select biologically important metalloproteins in gut microbes. Proteins that are conserved across multiple microbial taxa may perform essential functions for general bacterial survival in the human gut. Conversely, proteins that are unique to specific gut microbes may perform specialized functions, conferring a niche advantage to those bacteria in the gut habitat. Additionally, comparative genomics of closely related host-associated and non-host-associated strains may reveal proteins with specialized functions in the human gut. On the other hand, host factors can be used to select novel metalloenzymes for characterization. Integrated multi-omic methods can reveal differential presence or abundance of microbial metalloproteins correlated with disease state, diet, or therapeutic intervention. The incorporation of transcriptomic and proteomic data can reveal metalloproteins that are functionally active in different conditions. Ultimately, different rationales can guide selection and investigation of novel metalloenzyme functions.

Once individual metalloproteins or protein subfamilies have been identified and prioritized for study, the challenge remains to determine the biochemical function and substrate specificity. Bioinformatic tools, such as the EFI genome neighborhood network analysis [124, 125], rely on genomic localization and co-occurrence to develop rational hypotheses using the annotated functions of neighboring genes. However, due to the frequency of functional misannotation, experimental tools are needed to validate computationally predicted functions. Genetic knockouts are the gold standard for experimental function determination; however, genetic tools are largely lacking for human gut microbes. Recent efforts to develop new genetic tools for non-model organisms, such as *C. sporogenes* and *E. lenta* [135–137], will enable rigorous in vivo function discovery and validation in the future. Additionally, approaches for genetic manipulation within a microbial community will be invaluable for assessing the impact of metalloenzymes on community dynamics and host biology [138]. In the absence of genetic tools, other experimental strategies are required, such as in vitro biochemistry and integrated multi-omics. In vitro approaches often involve hypothesis-driven testing of substrate panels and monitoring activity by mass spectrometry. Alternatively, integrated multi-omic analyses (e.g. genomics combined with metabolomics) can provide an unbiased route to associate proteins with metabolic functions [86, 139]. This strategy has been used to overcome the challenges of cultivating certain human gut microbes in a laboratory setting. Ultimately, a combination of these approaches will reveal novel biochemical reactions catalyzed by metalloenzymes and improve microbial genome annotation.

New metalloenzymes discovered in the human gut microbiota are anticipated to have useful applications in biomedicine and biotechnology. As highlighted in this review, metalloenzymes play central roles in microbial metabolism that are intricately linked to human health. Therefore, by characterizing new enzymatic functions, we are likely to uncover molecular insights into host-microbe interactions and new targets for disease prevention and intervention. In addition to biomedical applications, gut microbial metalloenzymes perform challenging chemical transformations that could be used for biocatalytic and synthetic biology applications. Although oxygen-sensitive metalloproteins present unique challenges for biotechnological development [140], these obstacles are worth overcoming to utilize the catalytic power of these enzymes.

In conclusion, metalloenzymes serve key functions in the anaerobic metabolism of human gut microbes. They perform unique biochemical reactions that enable the utilization of various resources originating from the host, diet, and xenobiotic sources. Metalloenzymes significantly modify these substrates, influencing their bioactivity and bioavailability in the host. Critically, metabolic pathways involving metalloenzymes have strong associations with microbial pathogenesis and human disease. Thus, the discovery of novel metalloenzymes in human gut microbes offers exciting opportunities to define new chemistry for biomedical and biotechnological applications.

## Data Availability

The human gut microbial genomes analyzed in this article were accessed from the DOE Joint Genome Institute Integrated Microbial Genomes and Metagenomes database (https://img.jgi.doe.gov/). The data derived will be shared upon reasonable request to the corresponding author.

## References

[1] Qin J, Li Y, Cai Z et al. A metagenome-wide association study of gut microbiota in type 2 diabetes. Nature 2012;490:55–60. 10.1038/nature11450 23023125

[2] Nicholson JK, Holmes E, Kinross J et al. Host-gut microbiota metabolic interactions. Science 2012;336:1262–7. 10.1126/science.1223813 22674330

[3] David LA, Maurice CF, Carmody RN et al. Diet rapidly and reproducibly alters the human gut microbiome. Nature 2014;505:559–63. 10.1038/nature12820 24336217 PMC3957428

[4] Koppel N, Balskus EP. Exploring and understanding the biochemical diversity of the human microbiota. Cell Chem Biol 2016;23:18–30. 10.1016/j.chembiol.2015.12.008 26933733

[5] Chittim CL, Irwin SM, Balskus EP. Deciphering human gut microbiota—nutrient interactions: a role for biochemistry. Biochemistry 2018;57:2567–77. 10.1021/acs.biochem.7b01277 29669199

[6] Koppel N, Maini Rekdal V, Balskus EP. Chemical transformation of xenobiotics by the human gut microbiota. Science 2017;356:eaag2770. 10.1126/science.aag2770 28642381 PMC5534341

[7] Diener C, Dai CL, Wilmanski T et al. Genome—microbiome interplay provides insight into the determinants of the human blood metabolome. Nat Metab 2022; 4:1560–72, 10.1038/s42255-022-00670-1 36357685 PMC9691620

[8] Yatsunenko T, Rey FE, Manary MJ et al. Human gut microbiome viewed across age and geography. Nature 2012;486:222–7. 10.1038/nature11053 22699611 PMC3376388

[9] Lozupone CA, Stombaugh JI, Gordon JI et al. Diversity, stability and resilience of the human gut microbiota. Nature 2012;489:220–30. 10.1038/nature11550 22972295 PMC3577372

[10] Sharon G, Garg N, Debelius J et al. Specialized metabolites from the microbiome in health and disease. Cell Metab 2014;20:719–30. 10.1016/j.cmet.2014.10.016 25440054 PMC4337795

[11] Ursell LK, Haiser HJ, Van Treuren W et al. The intestinal metabolome: an intersection between microbiota and host. Gastroenterology 2014;146:1470–6. 10.1053/j.gastro.2014.03.001 24631493 PMC4102302

[12] Krautkramer KA, Fan J, Bäckhed F. Gut microbial metabolites as multi-kingdom intermediates. Nat Rev Micro 2021;19:77–94. 10.1038/s41579-020-0438-4 32968241

[13] Joice R, Yasuda K, Shafquat A et al. Determining microbial products and identifying molecular targets in the human microbiome. Cell Metab 2014;20:731–41. 10.1016/j.cmet.2014.10.003 25440055 PMC4254638

[14] Rajakovich LJ, Balskus EP. Metabolic functions of the human gut microbiota: the role of metalloenzymes. Nat Prod Rep 2019;36:593–625. 10.1039/c8np00074c 30452039 PMC7771511

[15] Espey MG . Role of oxygen gradients in shaping redox relationships between the human intestine and its microbiota. Free Radical Biol Med 2013;55:130–40. 10.1016/j.freeradbiomed.2012.10.554 23127782

[16] Beinert H . Iron-sulfur proteins: ancient structures, still full of surprises. J Biol Inorg Chem 2000;5:2–15. 10.1007/s007750050002 10766431

[17] Beinert H, Holm RH, Münck E. Iron-sulfur clusters: nature's modular, multipurpose structures. Science 1997;277:653–9. 10.1126/science.277.5326.653 9235882

[18] Johnson DC, Dean DR, Smith AD et al. Structure, function, and formation of biological iron-sulfur clusters. Annu Rev Biochem 2005;74:247–81. 10.1146/annurev.biochem.74.082803.133518 15952888

[19] Liu J, Chakraborty S, Hosseinzadeh P et al. Metalloproteins containing cytochrome, iron—sulfur, or copper redox centers. Chem Rev 2014;114:4366–469. 10.1021/cr400479b 24758379 PMC4002152

[20] Fuss JO, Tsai C-L, Ishida JP et al. Emerging critical roles of Fe—S clusters in DNA replication and repair. Biochim Biophys Acta (BBA)—Mol Cell Res 2015;1853:1253–71. 10.1016/j.bbamcr.2015.01.018 PMC4576882 25655665

[21] Stickland LH . Studies in the metabolism of the strict anaerobes (genus Clostridium): the chemical reactions by which Cl. sporogenes obtains its energy. Biochem J 1934;28:1746–59. 10.1042/bj0281746 16745572 PMC1253397

[22] Nisman B . The stickland reaction. Bacteriol Rev 1954;18:16–42. 10.1128/br.18.1.16-42.1954 13140081 PMC180783

[23] Buckel W, Martins BM, Messerschmidt A et al. Radical-mediated dehydration reactions in anaerobic bacteria. Biol Chem 2005;386:951–9. 10.1515/BC.2005.111 16218867

[24] Kim J, Hetzel M, Boiangiu CD et al. Dehydration of (*R*)-2-hydroxyacyl-CoA to enoyl-CoA in the fermentation of α-amino acids by anaerobic bacteria. FEMS Microbiol Rev 2004;28:455–68. 10.1016/j.femsre.2004.03.001 15374661

[25] Pavao A, Graham M, Arrieta-Ortiz ML et al. Reconsidering the *in vivo* functions of *Clostridial* Stickland amino acid fermentations. Anaerobe 2022;76:102600. 10.1016/j.anaerobe.2022.102600 35709938 PMC9831356

[26] de Vladar HP . Amino acid fermentation at the origin of the genetic code. Biol Direct 2012;7:6. 10.1186/1745-6150-7-6 22325238 PMC3376031

[27] Liu Y, Chen H, Van Treuren W et al. Clostridium sporogenes uses reductive Stickland metabolism in the gut to generate ATP and produce circulating metabolites. Nat Microbiol 2022;7:695–706. 10.1038/s41564-022-01109-9 35505245 PMC9089323

[28] Girinathan BP, DiBenedetto N, Worley JN et al. In vivo commensal control of *Clostridioides difficile* virulence. Cell Host Microbe 2021;29:1693–1708.e7. 10.1016/j.chom.2021.09.007 34637781 PMC8651146

[29] Fletcher JR, Erwin S, Lanzas C et al. Shifts in the gut metabolome and *Clostridium difficile* transcriptome throughout colonization and infection in a mouse model. mSphere 2018;3: 10.1128/msphere.00089–18. 10.1128/msphere.00089-18 PMC5874438 29600278

[30] Fletcher JR, Pike CM, Parsons RJ et al. Clostridioides difficile exploits toxin-mediated inflammation to alter the host nutritional landscape and exclude competitors from the gut microbiota. Nat Commun 2021;12:462. 10.1038/s41467-020-20746-4 33469019 PMC7815924

[31] Hofmann JD, Otto A, Berges M et al. Metabolic reprogramming of *Clostridioides difficile* during the stationary phase with the induction of toxin production. Front Microbiol 2018;9:1–17. 10.3389/fmicb.2018.01970 30186274 PMC6110889

[32] Neumann-Schaal M, Hofmann JD, Will SE et al. Time-resolved amino acid uptake of *Clostridium difficile* 630Δerm and concomitant fermentation product and toxin formation. BMC Microbiol 2015;15:281. 10.1186/s12866-015-0614-2 26680234 PMC4683695

[33] Bouillaut L, Dubois T, Sonenshein AL et al. Integration of metabolism and virulence in *Clostridium difficile*. Res Microbiol 2015;166:375–83. 10.1016/j.resmic.2014.10.002 25445566 PMC4398617

[34] Lamas B, Richard ML, Leducq V et al. CARD9 impacts colitis by altering gut microbiota metabolism of tryptophan into aryl hydrocarbon receptor ligands. Nat Med 2016;22:598–605. 10.1038/nm.4102 27158904 PMC5087285

[35] Guo J, Wang P, Cui Y et al. Protective effects of hydroxyphenyl propionic acids on lipid metabolism and gut microbiota in mice fed a high-fat diet. Nutrients 2023;15:1043. 10.3390/nu15041043 36839401 PMC9959022

[36] Wlodarska M, Luo C, Kolde R et al. Indoleacrylic acid produced by commensal peptostreptococcus species suppresses inflammation. Cell Host Microbe 2017;22:25–37.e6. 10.1016/j.chom.2017.06.007 28704649 PMC5672633

[37] Venkatesh M, Mukherjee S, Wang H et al. Symbiotic bacterial metabolites regulate gastrointestinal barrier function via the xenobiotic sensor PXR and toll-like receptor 4. Immunity 2014;41:296–310. 10.1016/j.immuni.2014.06.014 25065623 PMC4142105

[38] Hu J, Chen J, Xu X et al. Gut microbiota-derived 3-phenylpropionic acid promotes intestinal epithelial barrier function via AhR signaling. Microbiome 2023;11:102. 10.1186/s40168-023-01551-9 37158970 PMC10165798

[39] Zhao N, Liu C, Li N et al. Role of Interleukin-22 in ulcerative colitis. Biomed Pharmacother 2023;159:114273. 10.1016/j.biopha.2023.114273 36696801

[40] Knauer SH, Buckel W, Dobbek H. Structural basis for reductive radical formation and electron recycling in (R)-2-hydroxyisocaproyl-CoA dehydratase. J Am Chem Soc 2011;133:4342–7. 10.1021/ja1076537 21366233

[41] Buckel W, Zhang J, Friedrich P et al. Enzyme catalyzed radical dehydrations of hydroxy acids. Biochim Biophys Acta (BBA)—Proteins Proteom 2012;1824:1278–90. 10.1016/j.bbapap.2011.11.009 22178228

[42] Hans M, Buckel W, Bill E. The iron—sulfur clusters in 2-hydroxyglutaryl-CoA dehydratase from *Acidaminococcus fermentans*. Eur J Biochem 2000;267:7082–93. 10.1046/j.1432-1327.2000.01809.x 11106419

[43] Dickert S, Pierik AJ, Buckel W. Molecular characterization of phenyllactate dehydratase and its initiator from *Clostridium sporogenes*: Clostridial phenyllactate dehydratase. Mol Microbiol 2002;44:49–60. 10.1046/j.1365-2958.2002.02867.x 11967068

[44] Buckel W, Hetzel M, Kim J. ATP-driven electron transfer in enzymatic radical reactions. Curr Opin Chem Biol 2004;8:462–7. 10.1016/j.cbpa.2004.07.001 15450487

[45] Knauer SH, Buckel W, Dobbek H. On the ATP-dependent activation of the radical enzyme (R)-2-hydroxyisocaproyl-CoA dehydratase. Biochemistry 2012;51:6609–22. 10.1021/bi300571z 22827463

[46] Buckel W . Enzymatic reactions involving ketyls: from a chemical curiosity to a general biochemical mechanism. Biochemistry 2019;58:5221–33. 10.1021/acs.biochem.9b00171 30995029

[47] Kim J, Darley DJ, Buckel W et al. An allylic ketyl radical intermediate in clostridial amino-acid fermentation. Nature 2008;452:239–42. 10.1038/nature06637 18337824

[48] Kim J, Darley D, Buckel W. 2-Hydroxyisocaproyl-CoA dehydratase and its activator from *Clostridium difficile*: 2-Hydroxyisocaproyl-CoA dehydratase. FEBS J 2005;272:550–61. 10.1111/j.1742-4658.2004.04498.x 15654892

[49] Buckel W, Golding BT. Radical enzymes in anaerobes. Annu Rev Microbiol 2006;60:27–49. 10.1146/annurev.micro.60.080805.142216 16704345

[50] Broderick JB, Duffus BR, Duschene KS et al. Radical S-adenosylmethionine enzymes. Chem Rev 2014;114:4229–317. 10.1021/cr4004709 24476342 PMC4002137

[51] Broderick JB, Broderick WE, Hoffman BM. Radical SAM enzymes: nature's choice for radical reactions. FEBS Lett 2023;597:92–101. 10.1002/1873-3468.14519 36251330 PMC9894703

[52] Frey PA, Hegeman AD, Ruzicka FJ. The radical SAM superfamily. Crit Rev Biochem Mol Biol 2008;43:63–88. 10.1080/10409230701829169 18307109

[53] Vey JL, Yang J, Li M et al. Structural basis for glycyl radical formation by pyruvate formate-lyase activating enzyme. Proc Natl Acad Sci USA 2008;105:16137–41. 10.1073/pnas.0806640105 18852451 PMC2571006

[54] Yang H, McDaniel EC, Impano S et al. The elusive 5′-deoxyadenosyl radical: captured and characterized by electron paramagnetic resonance and electron nuclear double resonance spectroscopies. J Am Chem Soc 2019;141:12139–46. 10.1021/jacs.9b05926 31274303 PMC6784836

[55] Sayler RI, Stich TA, Joshi S et al. Trapping and electron paramagnetic resonance characterization of the 5′dAdo• radical in a radical S-adenosyl methionine enzyme reaction with a non-native substrate. ACS Cent Sci 2019;5:1777–85. 10.1021/acscentsci.9b00706 31807679 PMC6891858

[56] Hoffman BM, Broderick WE, Broderick JB. Mechanism of radical initiation in the radical SAM enzyme superfamily. Annu Rev Biochem 2023;92:333–49; null. 10.1146/annurev-biochem-052621-090638 37018846 PMC10759928

[57] Horitani M, Shisler K, Broderick WE et al. Radical SAM catalysis via an organometallic intermediate with an Fe—[5′-C]-deoxyadenosyl bond. Science 2016;352:822–5. 10.1126/science.aaf5327 27174986 PMC4929858

[58] Byer AS, Yang H, McDaniel EC et al. Paradigm shift for radical S-adenosyl-l-methionine reactions: the organometallic intermediate Ω is central to catalysis. J Am Chem Soc 2018;140:8634–8. 10.1021/jacs.8b04061 29954180 PMC6053644

[59] Shisler KA, Broderick JB. Glycyl radical activating enzymes: structure, mechanism, and substrate interactions. Arch Biochem Biophys 2014;546:64–71. 10.1016/j.abb.2014.01.020 24486374 PMC4083501

[60] Sawers RG, Clark DP. Fermentative pyruvate and Acetyl-Coenzyme A. EcoSal Plus 2004;1:1–37. 10.1128/ecosalplus.3.5.3. 10.1128/ecosalplus.3.5.3 26443368

[61] Nordlund P, Reichard P. Ribonucleotide reductases. Annu Rev Biochem 2006;75:681–706. 10.1146/annurev.biochem.75.103004.142443 16756507

[62] Backman LRF, Funk MA, Dawson CD et al. New tricks for the glycyl radical enzyme family. Crit Rev Biochem Mol Biol 2017;52:674–95. 10.1080/10409238.2017.1373741 28901199 PMC5911432

[63] Craciun S, Balskus EP. Microbial conversion of choline to trimethylamine requires a glycyl radical enzyme. Proc Natl Acad Sci USA 2012;109:21307–12. 10.1073/pnas.1215689109 23151509 PMC3535645

[64] Martínez-del Campo A, Bodea S, Hamer HA et al. Characterization and detection of a widely distributed gene cluster that predicts anaerobic choline utilization by human gut bacteria. mBio 2015;6:e00042–15. 10.1128/mBio.00042-15 25873372 PMC4453576

[65] Mitchell SC, Smith RL. Trimethylaminuria: the fish malodor syndrome. Drug Metab Dispos Biol Fate Chem 2001;29:517–21. 11259343

[66] Lang D, Yeung C, Peter R et al. Isoform specificity of trimethylamine N-oxygenation by human flavin-containing monooxygenase (FMO) and P450 enzymes. Biochem Pharmacol 1998;56:1005–12. 10.1016/S0006-2952(98)00218-4 9776311

[67] Tang WHW, Hazen SL. The contributory role of gut microbiota in cardiovascular disease. J Clin Invest 2014;124:4204–11. 10.1172/JCI72331 25271725 PMC4215189

[68] Zeisel SH, Warrier M. Trimethylamine *N*-oxide, the microbiome, and heart and kidney disease. Annu Rev Nutr 2017;37:157–81. 10.1146/annurev-nutr-071816-064732 28715991

[69] Chen Y, Liu Y, Zhou R et al. Associations of gut-flora-dependent metabolite trimethylamine-N-oxide, betaine and choline with non-alcoholic fatty liver disease in adults. Sci Rep 2016;6:19076. 10.1038/srep19076 26743949 PMC4705470

[70] Levin BJ, Huang YY, Peck SC et al. A prominent glycyl radical enzyme in human gut microbiomes metabolizes *trans*-4-hydroxy-l-proline. Science 2017;355:eaai8386. 10.1126/science.aai8386 28183913 PMC5705181

[71] Huang YY, Martínez-del Campo A, Balskus EP. Anaerobic 4-hydroxyproline utilization: discovery of a new glycyl radical enzyme in the human gut microbiome uncovers a widespread microbial metabolic activity. Gut Microbes 2018;9:1–16. 10.1080/19490976.2018.1435244 PMC6219649 29405826

[72] Jackson S, Calos M, Myers A et al. Analysis of proline reduction in the nosocomial pathogen *Clostridium difficile*. J Bacteriol 2006;188:8487–95. 10.1128/jb.01370-06 17041035 PMC1698225

[73] Reed AD, Fletcher JR, Huang YY et al. The Stickland reaction precursor trans-4-hydroxy-l-proline differentially impacts the metabolism of *Clostridioides difficile* and commensal clostridia. mSphere 2022;7:e00926–21. 10.1128/msphere.00926-21 35350846 PMC9044972

[74] Selmer T, Andrei PI. p*-*Hydroxyphenylacetate decarboxylase from *Clostridium difficile*: a novel glycyl radical enzyme catalysing the formation of *p*-cresol. Eur J Biochem 2001;268:1363–72. 10.1046/j.1432-1327.2001.02001.x 11231288

[75] Dawson LF, Stabler RA, Wren BW. Assessing the role of p-cresol tolerance in *Clostridium difficile*. J Med Microbiol 2008;57:745–9. 10.1099/jmm.0.47744-0 18480332

[76] Dawson LF, Donahue EH, Cartman ST et al. The analysis of para-cresol production and tolerance in *Clostridium difficile* 027 and 012 strains. BMC Microbiol 2011;11:86. 10.1186/1471-2180-11-86 21527013 PMC3102038

[77] Karplus PA, Fox KM, Massey V. Structure-function relations for old yellow enzyme. FASEB J 1995;9:1518–26. 10.1096/fasebj.9.15.8529830 8529830

[78] Hall B, Levy S, Dufault-Thompson K et al. BilR is a gut microbial enzyme that reduces bilirubin to urobilinogen. Nat Microbiol 2024;9:173–84. 10.1038/s41564-023-01549-x 38172624 PMC10769871

[79] Hubbard PA, Liang X, Schulz H et al. The crystal structure and reaction mechanism of *Escherichia coli* 2,4-dienoyl-CoA reductase. J Biol Chem 2003;278:37553–60. 10.1074/jbc.M304642200 12840019

[80] Pascal Andreu V, Fischbach MA, Medema MH. Computational genomic discovery of diverse gene clusters harbouring Fe-S flavoenzymes in anaerobic gut microbiota. Microb Genomics 2020;6:1–8. 10.1099/mgen.0.000373 PMC7371122 32416747

[81] Wahlström A, Sayin SI, Marschall H-U et al. Intestinal crosstalk between bile acids and microbiota and its impact on host metabolism. Cell Metab 2016;24:41–50. 10.1016/j.cmet.2016.05.005 27320064

[82] Collins SL, Stine JG, Bisanz JE et al. Bile acids and the gut microbiota: metabolic interactions and impacts on disease. Nat Rev Micro 2023;21:236–47. 10.1038/s41579-022-00805-x PMC12536349 36253479

[83] Jia W, Xie G, Jia W. Bile acid—microbiota crosstalk in gastrointestinal inflammation and carcinogenesis. Nat Rev Gastroenterol Hepatol 2018;15:111–28. 10.1038/nrgastro.2017.119 29018272 PMC5899973

[84] Ridlon JM, Kang D-J, Hylemon PB. Bile salt biotransformations by human intestinal bacteria. J Lipid Res 2006;47:241–59. 10.1194/jlr.R500013-JLR200 16299351

[85] Funabashi M, Grove TL, Wang M et al. A metabolic pathway for bile acid dehydroxylation by the gut microbiome. Nature 2020;582:566–70. 10.1038/s41586-020-2396-4 32555455 PMC7319900

[86] Kenny DJ, Plichta DR, Shungin D et al. Cholesterol metabolism by uncultured human gut bacteria influences host cholesterol level. Cell Host Microbe 2020;28:245–257.e6. 10.1016/j.chom.2020.05.013 32544460 PMC7435688

[87] Kapitulnik J . Bilirubin: an endogenous product of heme degradation with both cytotoxic and cytoprotective properties. Mol Pharmacol 2004;66:773–9. 10.1124/mol.104.002832 15269289

[88] Stenemo M, Ganna A, Salihovic S et al. The metabolites urobilin and sphingomyelin (30:1) are associated with incident heart failure in the general population. ESC Heart Fail 2019;6:764–73. 10.1002/ehf2.12453 31148414 PMC6676274

[89] Kipp ZA, Xu M, Bates EA et al. Bilirubin levels are negatively correlated with adiposity in obese men and women, and its catabolized product, urobilin, is positively associated with insulin resistance. Antioxidants 2023;12:170. 10.3390/antiox12010170 36671031 PMC9854555

[90] Panche AN, Diwan AD, Chandra SR. Flavonoids: an overview. J Nutr Sci 2016;5:e47. 10.1017/jns.2016.41 28620474 PMC5465813

[91] Murota K, Nakamura Y, Uehara M. Flavonoid metabolism: the interaction of metabolites and gut microbiota. Biosci Biotechnol Biochem 2018;82:600–10. 10.1080/09168451.2018.1444467 29504827

[92] Jackson RL, Greiwe JS, Schwen RJ. Emerging evidence of the health benefits of S-equol, an estrogen receptor β agonist. Nutr Rev 2011;69:432–48. 10.1111/j.1753-4887.2011.00400.x 21790611

[93] Schröder C, Matthies A, Engst W et al. Identification and expression of genes involved in the conversion of daidzein and genistein by the equol-forming bacterium Slackia isoflavoniconvertens. Appl Environ Microb 2013;79:3494–502. 10.1128/AEM.03693-12 PMC3648055 23542626

[94] Tsuji H, Moriyama K, Nomoto K et al. Identification of an enzyme system for daidzein-to-equol conversion in Slackia sp. Appl Environ Microb 2012;78:1228–36. 10.1128/AEM.06779-11 PMC3272994 22179235

[95] Pelosi L, Vo CDT, Abby SS et al. Ubiquinone biosynthesis over the entire O_2_ range: characterization of a conserved O_2_-independent pathway. mBio 2019;10:1–21. 10.1128/MBIO.01319-19/FORMAT/EPUB PMC6747719 31289180

[96] Lauhon CT . Identification and characterization of genes required for 5-hydroxyuridine synthesis in *Bacillus subtilis* and *Escherichia coli* tRNA. J Bacteriol 2019;201:1–18. 10.1128/JB.00433-19 PMC6755726 31358606

[97] Kimura S, Sakai Y, Ishiguro K et al. Biogenesis and iron-dependency of ribosomal RNA hydroxylation. Nucleic Acids Res 2017;45:12974–86. 10.1093/nar/gkx969 29069499 PMC5727448

[98] Sakai Y, Kimura S, Suzuki T. Dual pathways of tRNA hydroxylation ensure efficient translation by expanding decoding capability. Nat Commun 2019;10:1–16. 10.1038/s41467-019-10750-8 31253794 PMC6599085

[99] Søballe B, Poole RK. Microbial ubiquinones: multiple roles in respiration, gene regulation and oxidative stress management. Microbiology 1999;145:1817–30. 10.1099/13500872-145-8-1817 10463148

[100] Arias-Cartin R, Kazemzadeh Ferizhendi K, Séchet E et al. Role of the *Escherichia coli* ubiquinone-synthesizing UbiUVT pathway in adaptation to changing respiratory conditions. mBio 2023;0:e03298–22. 10.1128/mbio.03298-22 PMC10470549 37283518

[101] Vo C-D-T, Michaud J, Elsen S et al. The O2-independent pathway of ubiquinone biosynthesis is essential for denitrification in Pseudomonas aeruginosa. J Biol Chem 2020;295:9021–32. 10.1074/jbc.RA120.013748 32409583 PMC7335794

[102] Chionh YH, McBee M, Babu IR et al. tRNA-mediated codon-biased translation in mycobacterial hypoxic persistence. Nat Commun 2016;7:13302. 10.1038/ncomms13302 27834374 PMC5114619

[103] Schacherl M, Montada AaM, Brunstein E et al. The first crystal structure of the peptidase domain of the U32 peptidase family. Acta Crystallogr D Biol Crystallogr 2015;71:2505–12. 10.1107/S1399004715019549 26627657

[104] Hille R . Molybdenum-containing hydroxylases. Arch Biochem Biophys 2005;433:107–16. 10.1016/j.abb.2004.08.012 15581570

[105] Li B, Bridwell-Rabb J. Aerobic enzymes and their radical SAM enzyme counterparts in tetrapyrrole pathways. Biochemistry 2019;58:85–93. 10.1021/acs.biochem.8b00906 30365306

[106] Ferizhendi KK, Simon P, Pelosi L et al. An organic O donor for biological hydroxylation reactions. Proc Natl Acad Sci USA 2024;121:e2321242121. 10.1073/pnas.2321242121 38507448 PMC10990095

[107] Rao G, Oldfield E. Structure and function of four classes of the 4Fe—4S protein, IspH. Biochemistry 2016;55:4119–29. 10.1021/acs.biochem.6b00474 27357244 PMC4961616

[108] Wang W, Wang K, Liu Y-L et al. Bioorganometallic mechanism of action, and inhibition, of IspH. Proc Natl Acad Sci USA 2010;107:4522–7. 10.1073/pnas.0911087107 20173096 PMC2842026

[109] Wang W, Oldfield E. Bioorganometallic chemistry with IspG and IspH: structure, function, and inhibition of the [Fe_4_S_4_] proteins involved in isoprenoid biosynthesis. Angew Chem Int Ed 2014;53:4294–310. 10.1002/anie.201306712 PMC3997630 24481599

[110] Hille R . The molybdenum oxotransferases and related enzymes. Dalton Trans 2013;42:3029. 10.1039/c2dt32376a 23318732

[111] Leimkühler S, Iobbi-Nivol C. Bacterial molybdoenzymes: old enzymes for new purposes. FEMS Microbiol Rev 2016;40:1–18. 10.1093/femsre/fuv043 26468212

[112] Little AS, Younker IT, Schechter MS et al. Dietary- and host-derived metabolites are used by diverse gut bacteria for anaerobic respiration. Nat Microbiol 2024;9:55–69. 10.1038/s41564-023-01560-2 38177297 PMC11055453

[113] Winter SE, Thiennimitr P, Winter MG et al. Gut inflammation provides a respiratory electron acceptor for Salmonella. Nature 2010;467:426–9. 10.1038/nature09415 20864996 PMC2946174

[114] Hinsley AP, Berks BC. Specificity of respiratory pathways involved in the reduction of sulfur compounds by Salmonella enterica. Microbiol Read Engl 2002;148:3631–8. 10.1099/00221287-148-11-3631 12427953

[115] Kisker C, Schindelin H, Rees DC. Molybdenum-cofactor—containing enzymes: structure and mechanism. Annu Rev Biochem 1997;66:233–67. 10.1146/annurev.biochem.66.1.233 9242907

[116] Le CC, Bae M, Kiamehr S et al. Emerging chemical diversity and potential applications of enzymes in the DMSO reductase superfamily. Annu Rev Biochem 2022;91:475–504. 10.1146/annurev-biochem-032620-110804 35320685

[117] Maini Rekdal V, Nol Bernadino P, Luescher MU et al. A widely distributed metalloenzyme class enables gut microbial metabolism of host- and diet-derived catechols. eLife 2020;9:e50845. 10.7554/eLife.50845 32067637 PMC7028382

[118] Maini Rekdal V, Bess EN, Bisanz JE et al. Discovery and inhibition of an interspecies gut bacterial pathway for Levodopa metabolism. Science 2019;364:eaau6323. 10.1126/science.aau6323 31196984 PMC7745125

[119] Eisenhofer G, Åneman A, Friberg P et al. Substantial production of dopamine in the human gastrointestinal tract. J Clin Endocrinol Metab 1997;82:3864–71. 10.1210/jcem.82.11.4339 9360553

[120] Li ZS, Schmauss C, Cuenca A et al. Physiological modulation of intestinal motility by enteric dopaminergic neurons and the D2 receptor: analysis of dopamine receptor expression, location, development, and function in wild-type and knock-out mice. J Neurosci 2006;26:2798–807. 10.1523/JNEUROSCI.4720-05.2006 16525059 PMC6675162

[121] Dichtl S, Demetz E, Haschka D et al. Dopamine is a siderophore-like iron chelator that promotes *Salmonella enterica* serovar typhimurium virulence in mice. mBio 2019;10:e02624–18. 10.1128/mBio.02624-18 30723125 PMC6428752

[122] Bess EN, Bisanz JE, Yarza F et al. Genetic basis for the cooperative bioactivation of plant lignans by *Eggerthella lenta* and other human gut bacteria. Nat Microbiol 2020;5:56–66. 10.1038/s41564-019-0596-1 31686027 PMC6941677

[123] McCurry MD, D'Agostino GD, Walsh JT et al. Gut bacteria convert glucocorticoids into progestins in the presence of hydrogen gas. Cell 2024;187:2952–2968.e13. 10.1016/j.cell.2024.05.005 38795705 PMC11179439

[124] Zallot R, Oberg N, Gerlt JA. The EFI web resource for genomic enzymology tools: leveraging protein, genome, and metagenome databases to discover novel enzymes and metabolic pathways. Biochemistry 2019;58:4169–82. 10.1021/acs.biochem.9b00735 31553576 PMC7057060

[125] Oberg N, Zallot R, Gerlt JA. EFI-EST, EFI-GNT, and EFI-CGFP: Enzyme Function Initiative (EFI) web resource for genomic enzymology tools. J Mol Biol 2023;435:168018. 10.1016/j.jmb.2023.168018 37356897 PMC10291204

[126] Oberg N, Precord TW, Mitchell DA et al. RadicalSAM.org: a resource to interpret sequence-function space and discover new radical SAM enzyme chemistry. ACS Bio Med Chem Au 2022;2:22–35. 10.1021/acsbiomedchemau.1c00048 PMC9477430 36119373

[127] Jumper J, Evans R, Pritzel A et al. Highly accurate protein structure prediction with AlphaFold. Nature 2021;596:583–9. 10.1038/s41586-021-03819-2 34265844 PMC8371605

[128] Varadi M, Anyango S, Deshpande M et al. AlphaFold Protein Structure Database: massively expanding the structural coverage of protein-sequence space with high-accuracy models. Nucleic Acids Res 2022;50:D439—D444. 10.1093/nar/gkab1061 34791371 PMC8728224

[129] Abramson J, Adler J, Dunger J et al. Accurate structure prediction of biomolecular interactions with AlphaFold 3. Nature 2024;630:493–500. 10.1038/s41586-024-07487-w 38718835 PMC11168924

[130] Hekkelman ML, de Vries I, Joosten RP et al. AlphaFill: enriching AlphaFold models with ligands and cofactors. Nat Methods 2023;20:205–13, 10.1038/s41592-022-01685-y 36424442 PMC9911346

[131] Cheng Y, Wang H, Xu H et al. Co-evolution-based prediction of metal-binding sites in proteomes by machine learning. Nat Chem Biol 2023;19:548–55. 10.1038/s41589-022-01223-z 36593274

[132] Gligorijević V, Renfrew PD, Kosciolek T et al. Structure-based protein function prediction using graph convolutional networks. Nat Commun 2021;12:3168. 10.1038/s41467-021-23303-9 34039967 PMC8155034

[133] Bak DW, Weerapana E. Monitoring Fe—S cluster occupancy across the *E. coli* proteome using chemoproteomics. Nat Chem Biol 2023;19:356–66. 10.1038/s41589-022-01227-9 36635565 PMC9992348

[134] Zeng X, Wei T, Wang X et al. Discovery of metal-binding proteins by thermal proteome profiling. Nat Chem Biol 2024;20, 770–8. 10.1038/s41589-024-01563-y 38409364

[135] Guo C-J, Allen BM, Hiam KJ et al. Depletion of microbiome-derived molecules in the host using Clostridium genetics. Science 2019;366:eaav1282. 10.1126/science.aav1282 31831639 PMC7141153

[136] Dong X, Guthrie BGH, Alexander M et al. Genetic manipulation of the human gut bacterium *Eggerthella lenta* reveals a widespread family of transcriptional regulators. Nat Commun 2022;13:7624. 10.1038/s41467-022-33576-3 36494336 PMC9734109

[137] Bisanz JE, Soto-Perez P, Noecker C et al. A genomic toolkit for the mechanistic dissection of intractable human gut bacteria. Cell Host Microbe 2020;27:1001–1013.e9. 10.1016/j.chom.2020.04.006 32348781 PMC7292766

[138] Rubin BE, Diamond S, Cress BF et al. Species- and site-specific genome editing in complex bacterial communities. Nat Microbiol 2022;7:34–47. 10.1038/s41564-021-01014-7 34873292 PMC9261505

[139] Mehta RS, Mayers JR, Zhang Y et al. Gut microbial metabolism of 5-ASA diminishes its clinical efficacy in inflammatory bowel disease. Nat Med 2023;29:700–9. 10.1038/s41591-023-02217-7 36823301 PMC10928503

[140] Jäger CM, Croft AK. If it is hard, it is worth doing: engineering radical enzymes from anaerobes. Biochemistry 2023;62, 241–52, 10.1021/acs.biochem.2c00376 36121716 PMC9850924

